# Development of Polymer-Based Nanoformulations for Glioblastoma Brain Cancer Therapy and Diagnosis: An Update

**DOI:** 10.3390/polym13234114

**Published:** 2021-11-26

**Authors:** Bijuli Rabha, Kaushik Kumar Bharadwaj, Siddhartha Pati, Bhabesh Kumar Choudhury, Tanmay Sarkar, Zulhisyam Abdul Kari, Hisham Atan Edinur, Debabrat Baishya, Leonard Ionut Atanase

**Affiliations:** 1 Department of Bioengineering & Technology, GUIST, Gauhati University, Guwahati 781014, India; bijulipep@gmail.com (B.R.); kkbhrdwj01@gmail.com (K.K.B.); 2 Skills Innovation & Academic Network (SIAN) Institute-Association for Biodiversity Conservation and Research (ABC), Balasore 756001, India; patisiddhartha@gmail.com; 3 NatNov Bioscience Private Limited, Balasore, 756001, India; 4 Department of Chemistry, Gauhati University, Guwahati 781014, India; bkcsat@gmail.com; 5 Malda Polytechnic, West Bengal State Council of Technical Education, Govt. of West Bengal, Malda 732102, India; tanmays468@gmail.com; 6 Department of Food Technology and Biochemical Engineering, Jadavpur University, Kolkata 700032, India; 7 Faculty of Agro Based Industry, Universiti Malaysia Kelantan, Jeli 17600, Malaysia; zulhisyam.a@umk.edu.my; 8 School of Health Sciences, Health Campus, Universiti Sains Malaysia, Kubang Kerian 16150, Malaysia; edinur@usm.my; 9 Faculty of Medical Dentistry, “Apollonia” University of Iasi, 700511 Iasi, Romania

**Keywords:** polymer nanoparticles, glioma/glioblastoma, blood–brain barrier (BBB)/blood brain tumour barrier (BBTB), nanodiagnostics, drug delivery and imaging

## Abstract

Brain cancers, mainly high-grade gliomas/glioblastoma, are characterized by uncontrolled proliferation and recurrence with an extremely poor prognosis. Despite various conventional treatment strategies, viz., resection, chemotherapy, and radiotherapy, the outcomes are still inefficient against glioblastoma. The blood–brain barrier is one of the major issues that affect the effective delivery of drugs to the brain for glioblastoma therapy. Various studies have been undergone in order to find novel therapeutic strategies for effective glioblastoma treatment. The advent of nanodiagnostics, i.e., imaging combined with therapies termed as nanotheranostics, can improve the therapeutic efficacy by determining the extent of tumour distribution prior to surgery as well as the response to a treatment regimen after surgery. Polymer nanoparticles gain tremendous attention due to their versatile nature for modification that allows precise targeting, diagnosis, and drug delivery to the brain with minimal adverse side effects. This review addresses the advancements of polymer nanoparticles in drug delivery, diagnosis, and therapy against brain cancer. The mechanisms of drug delivery to the brain of these systems and their future directions are also briefly discussed.

## 1. Introduction

Cancer is one of the serious life-threatening diseases worldwide with a higher risk of mortality, around 10 million new cases are diagnosed every year [1,2]. Among different types of cancer, brain cancer is the most lethal and invasive type of central nervous system (CNS) disorder [3]. Brain cancer is characterised as a heterogeneous group of primary and metastatic cancers in the CNS [4,5]. The average incidence of both malignant and non-malignant brain cancer is reported approximately 28.57 per 100,000 population, mostly affecting 0 to 19 years, with a mean annual morbidity rate of 5.57 per 100,000 population [6,7]. Among these, the malignant primary brain cancers with a 5-year survival rate of less than 33.3–35% and even the rate are still alleviating. The average survival span is still not improved and even lower between 15 to 22 months [8,9]. A recent report from 2020 of the Central Brain Tumor Registry of the United States accounted for primary malignant tumour incidence rate to be 7.08 per 100,000, with 123,484 estimated cases, and 16.71 per 100,000, with 291,927 cases of non-malignant tumour [10]. Malignant primary tumours, i.e., gliomas derived from the glial origin, are newly diagnosed for approximately 70%, mostly in adults [5,11]. The reduced efficacy of brain cancer therapy is mainly attributed to the presence of the blood–brain barrier (BBB) that limits the permeation of systemically applied drugs into the brain [3].

Brain cancers are categorised into two groups, viz., primary brain cancer originated from the brain and resided within the brain, commonly called glioma, and secondary or metastatic brain cancer spreading from primary cancer outside the CNS, originate from systemic neoplasms and further evolved in the interior of brain parenchyma [12,13]. Glial cell originated gliomas include glioblastomas, astrocytomas, schwannomas, oligodendrogliomas, etc. [14]. According to World Health Organization (WHO), glioma tumours of CNS is classified into four grades based on aggressiveness, Grade I pilocytic astrocytoma, Grade II diffuse astrocytoma, Grade III anaplastic astrocytoma, and Grade IV glioblastoma [12]. Glioblastoma (GBM) and its variants were categorised as Grade IV tumours [15]. Grades I and II are considered low-grade glioma, and Grades III and IV are considered high-grade gliomas, i.e., malignant gliomas, and are characterised by poor prognosis [8,16,17]. GBM can either develop from normal brain cells or evolve from pre-existing low-grade astrocytoma [18]. GBM is also termed as glioblastoma multiforme or Grade IV astrocytoma [19]. Excessive penetration and vascular proliferation into brain parenchyma is the indication of aggressive cancer [20].

Conventional glioma therapy includes tumour resection followed by radiotherapy and chemotherapy. Surgical resection is generally considered a standard method for glioblastoma therapy. Yet resection of tumour tissue cannot be entirely removed and hence is limited by the glioblastoma’s aggressiveness caused by penetration into surrounding tissue microenvironment and tumour vascularisation [20,21]. Hence, tumour resection is associated with the administration of chemotherapeutic drugs and/or radiation therapy for enhanced efficiency. Radiation therapy can be delivered internally or externally and is regarded as the standard treatment for high-grade gliomas [22]. Chemotherapy drugs such as carmustine (BCNU) can cross the BBB and target glioma cells directly [20]. Further, chemotherapy has undergone some alteration by replacing the use of some alkylating agents, viz., carmustine (BCNU), nimustine (ACNU), and lomustine (CCNU) with temozolomide (TMZ) [23]. Temozolomide is converted to 5-3-(methyl)-1-(triazen-1-yl) imidazole-4-carboxamide, at physiological pH, damages DNA via methylation of the O6-position of guanines, blocks DNA replication and induces tumour cell death. Presently, TMZ, along with surgical resection and radiotherapy, is applied for glioblastoma therapy [17]. Despite that, all the treatment strategies possess some limitations towards survival and thus, the prognosis still remains poor (Table 1).

Although brain cancer resembles to other forms of cancer in the body, the major difference is their intracranial neoplasms, heterogeneity, intricate brain system, and the physiological features of the cranial cavity which restrain the treatment options [10]. Gliomas tend to permeate the surrounding tissue microenvironment, and thereby, it is very difficult to determine the tumour boundaries. This also attributes to several difficulties in conventional therapeutic approaches for a curative outcome. Moreover, the physical and chemical barriers hamper therapeutic drug molecules from reaching tumour locations [11]. The BBB and blood–brain tumour barrier (BBTB) represent the diffusion barrier systems of the brain that regulate the influx of drugs to the brain except owing to certain characteristics [24]. Standard treatments remain ineffective due to poor surgical resection of tumours, mainly the infiltrative ones, poor chemo-therapeutic drug influx to the tumour site, and BBB that restrict them from diffusing toward tumour location [25]. The limitations of radiotherapy also result in incomplete eradication of GBM cells resulting in self-renewal and recurrence [26]. Targeting active anticancer agents to the brain is a challenging task in the area of drug delivery as BBB prevents the transportation of a drug. Hence, higher doses are needed to attain desired therapeutic efficacy which causes undesirable side effects [27].

## 2. The Blood–Brain Barrier (BBB)

**Figure 1:**
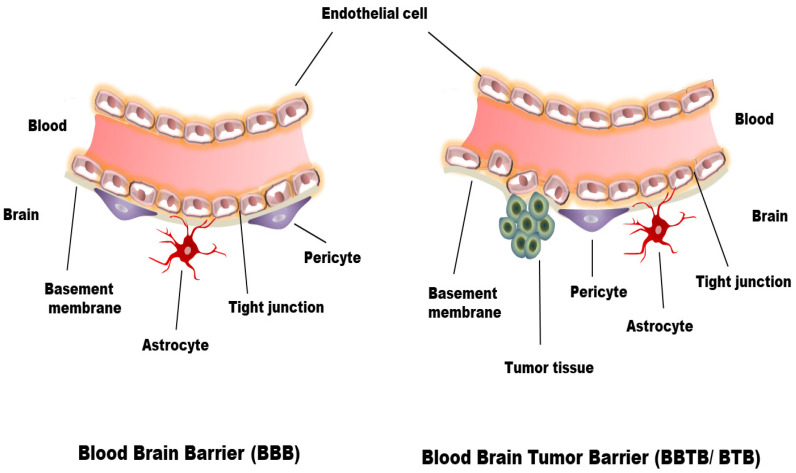
Schematic representation of blood–brain Barrier (BBB) and the blood–brain tumour barrier (BBTB).

One of the main hurdles for the effective systemic treatment of brain cancer is the presence of the BBB. The BBB is a semipermeable membrane barrier between blood capillaries and cellular components of brain tissues that control the movement of ions, nutrients, and cells. The BBB also serves for the dynamic transport of nutrients, peptides, proteins and immune cells between the brain and blood [28]. The BBB consists of endothelial cells, glial cells (pericytes, astrocytes, and neurons) and basement membrane [29] (Figure 1). The endothelial cells line the interior brain capillaries forming the tight junctions that allow small molecules, gases and curb the influx of harmful toxins or pathogens such as bacteria, lipophilic neurotoxins, xenobiotics and hydrophilic substances from the blood to the brain [30]. Due to the presence of pinocytic vesicles, other carriers, transport proteins, and large numbers of mitochondria, hydrophobic and essential molecules such as O_2_, CO_2_, glucose, hormones, etc. can infiltrate either by passive diffusion or active transport mechanisms [29]. The presence of several transmembrane proteins characterises the tight junctions between the inter-endothelial cells. These protein complexes are mainly comprised of occludin, claudin, and junctional adhesion molecules. These three specialised proteins interact to develop an intricate, tight barrier that is exclusive to the cerebro-endothelial cells [31]. The apical part of the endothelial cell is exposed to the brain’s blood capillaries, and the basolateral part is exposed to the cerebrospinal fluid supported by the basement membrane. The basement membrane with 30–40 nm thickness consists of Type IV collagen, fibronectin, laminin, heparin sulfate proteoglycans and other extracellular matrix proteins that completely covers the endothelial cells and limits the movement of the solutes [29,31,32]. Approximately 98% of smaller molecular weight drugs and 100% of larger molecular weight drugs are reported for their inability to cross the intact BBB [33,34]. Under various brain-related pathological conditions, including brain cancers, glioma cells loose the structural integrity and the function of the BBB [35]. BBB is compromised in human glioma cells because of the leaky inter endothelial tight junction and poorly differentiated astrocytes that are unable to release essential components for BBB function [31,36]. In this case, it is termed as blood–brain tumour barrier (BBTB) or blood–tumour barrier (BTB) [14] (Figure 1).

In low-grade gliomas, the structure and function of the BBTB resemble normal BBB, while in hi-grade glioma, BBB is significantly altered, disrupted. Although the degree of BBB disruption varies from the tumour malignancy, low-grade glioma is still a hurdle to treat due to intact BBB. Despite high-grade gliomas, the structural disruption of their vascular density and integrity is negligible to drug permeability in tumour cells [37,38]. However, BBTB is more permeable than the BBB and allows heterogeneous permeability to drugs and other components. Thus, it is a more challenging task to combat the difficulties of brain cancer [39]. Therefore, along with the existing therapeutic regimen, new approaches are required to combat the BBB. To combat these difficulties, various techniques were developed, which are mostly invasive and cause serious side effects. Nanotechnology, especially use of polymer nanoparticles, helps address the major hurdle of glioma therapy non-invasively. Polymer nanoparticles aid in the targeting and delivery of potent drug molecules to the brain. In this review paper, we will briefly summarise the up-to-date existing therapies and diagnoses in brain cancer gliomas using polymer nanoparticles.

## 3. Polymer Nanoparticles for Drug Delivery Strategy to Overcome the BBB

The BBB is the main problem in the treatment of brain cancer glioma. The chemotherapeutic drugs are mostly ineffective due to limiting permeability to BBB as it allows to pass only low molecular weight (<500 Da), electrically neutral hydrophobic drugs with lipophilicity at log P 2–3 [11,36,40]. The majority of chemotherapeutic drugs are larger in size, ionic, hydrophilic molecules and thus cannot cross the BBB that is attributed to the requirement of a higher systemic dose that results in severe side effects [11]. To overcome these drawbacks, nanoparticles can be utilised for the controlled and sustained delivery of drugs. Biodegradable polymer nanoparticles are extensively studied systems in cancer drug delivery and therapy. These nanoparticles are also highly stable and can be tuned in order to obtain the desirable characteristics for a passive or an active targeting [41]. Polymer nanoparticles can induce selective toxicity and can load ample anticancer drugs or other molecules. Various biodegradable polymeric drug delivery systems include nanogels or hydrogels, poly(ε-caprolactone) (PCL), poly (lactic-co-glycolic acid) (PLGA), chitosan [42,43], dendrimers, etc. [44]. Due to versatile tuneable properties, these nanoparticles can open tight junctions of BBB, shield BBB limiting properties of anticancer drugs, release the drug in a sustainable manner, prolong the systemic circulation, and protect against enzymatic degradation [1,45].

Studies showed that Resveratrol loaded PLGA: D-α-tocopheryl polyethylene glycol 1000 succinate blend nanoparticles (RSV-PLGA-BNPs) displayed significant increasing cytotoxicity and enhanced cell penetration in C6 glioma cells. Haemocompatibility evaluation is one of the critical analyses of interaction between nanoparticles and various blood components that determine any adverse effect upon nanoparticle exposure to blood. The nanoparticles should not cause haemolysis during and after infusions. The haemocompatibility analysis of RSV-PLGA-BNPs revealed safe for i.v. administration. The nanoparticles exhibited prolonged systemic circulation up to 36 h. The nanoparticles also showed higher brain accumulation, suggesting a potential system for the betterment of systemic circulation and plasma half-life with a promising anticancer effect against glioma [1]. In another study, L-carnitine-conjugated PLGA NPs were developed to target glioma cells. These NPs were found to significantly cross the BBB and showed a potential anti-glioma effect [46]. Lactoferrin decorated PEG-PLGA NPs was developed for the delivery of shikonin and the treatment of gliomas [47]. Lactoferrin coating promotes internalisation across the BBB. In vitro and in vivo experiments showed the enhanced nanoparticle uptake and distribution of NPs in the brain with effective treatment of glioblastomas.

## 4. Polymer Nanoparticles for Anticancer Drug Delivery to the Brain: Mechanism

**Figure 2:**
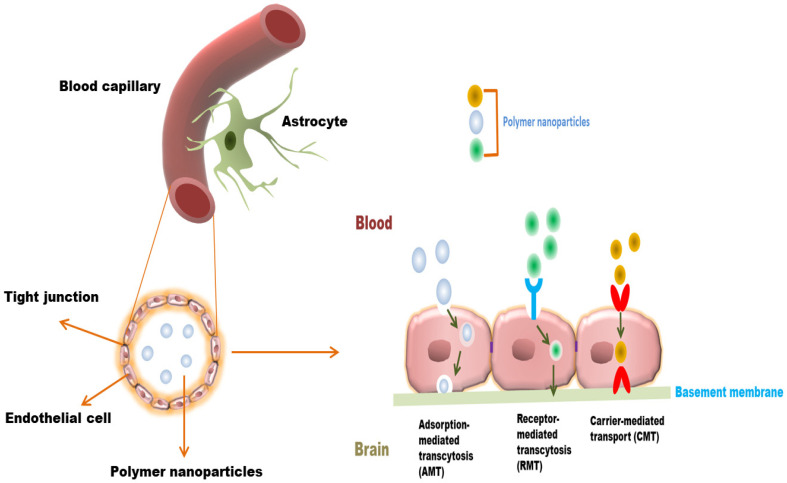
Various transport mechanisms of polymer NPs across blood–brain barrier (BBB).

Polymer nanoparticles can cross BBB or BBTB either passively or via active endocytosis mechanisms. The unmodified polymer NPs internalise BBB mainly through passive mechanism, the so-called enhanced permeability and retention (EPR) effect, which depends on nanoparticle size. However, the NPS internalised by a passive mechanism have comparatively lower brain uptake than ligand-functionalised polymer NPs [48]. Various strategies have been undertaken to improve the infiltration of NPs into the brain. These strategies involve modification of NPs with certain moieties or components to take benefit of BBB endocytosis pathways for drug delivery. Polymer nanoparticles are able to cross BBB/BBTB through adsorption-mediated transcytosis (AMT), carrier-mediated transport (CMT), and receptor-mediated transcytosis (RMT) [49,50,51,52] (Figure 2). The internalisation of polymer nanoparticles crossing BBB/BBTB is summarized in Table 2. Polymer nanoparticles with positively charged can electrostatically interact with a negatively charged luminal surface that is attributed to cross the BBB/BBTB. The cationic polymer nanoparticles can be achieved by various surface modification strategies, either by coating or conjugation of cationic polymer or surfactant to non-ionic or neutral polymer. These modifications of NPs have been shown to utilise the AMT mechanisms to improve brain uptake. For example, a study of cationic bovine serum albumin (CBSA) conjugated with poly (ethylene glycol)–b-poly(lactide) (PEG–PLA) nanoparticles (CBSA–NPs), loaded with 6-coumarin was reported for brain delivery. Results revealed that CBSA–NPs uptake in rat brain capillary endothelial cells (BCECs) was enhanced as compared to control group BSA conjugated with pegylated nanoparticles (BSA–NP) BSA–NPs. Fluorescent microscopy of coronal brain sections displayed increased accumulation of CBSA–NPs than of BSA–NPs [53].

In the CMT mechanism, polymers NPs are designed to deliver drugs in order to take advantage of carrier molecules present in BBB. Polymer NPs are modified or decorated with membrane-penetrating components such as amino acids, peptides, and nutrients capable of transporting cargo across the BBB endothelial cells by utilising systemic transporters. For example, 2-deoxy-D-glucose modified poly (ethylene glycol)-co-poly (trimethylene carbonate) nanoparticles (DGlu-NPs) were studied for targeting the glioma BBB. The internalisation of DGlu-NP on RG-2 rat glioma cells was significantly higher than that of non-modified nanoparticles. This was attributed to the recognition of NPs by GLUT1 leading to enhanced cellular internalisation in glioma cells than in surrounding normal tissue and thus exhibiting promising in vivo anti-glioma activity [54].

Similarly, L-carnitine modified PLGA nanoparticles (LC-PLGA NPs) were designed to utilise the advantage of Na-coupled carnitine transporter 2 (OCTN2) expressions on brain capillary endothelial cells as glioma cells for BBB infiltration and targeting. Results showed increased accumulation of NPs in the BBB endothelial cell line (hCMEC/D3) and the glioma cell line (T98G). This revealed the Na dependent cellular uptake that involves OCTN2 in the NPs internalisation process. Moreover, a higher accumulation of LC-PLGA NPs was also observed in the in vivo mouse model study. Furthermore, loading of drugs Taxol and paclitaxel in the LC-PLGA NPs improved anti-glioma activity in both 2D-cell and 3D-spheroid models [46].

With the RMT mechanism, polymer NPs are decorated/designed with targeting ligands that bind to specific cell surface receptors highly expressed in BBB transport pathways. For example, the Transferrin receptor (TfR) is one of the primary targets for investigating RMT across the BBB because of its high expression on BBB/BBTB endothelium [55]. To evaluate in vivo BBB penetration and targeting efficacy, transferrin modified doxorubicin (DOX) and paclitaxel (PTX) loaded magnetic silica PLGA nanoparticles (MNP-MSN-PLGA-Tf NPs) were developed. The nanoparticles were effectively accumulated in the tumour bearing mice suggesting that Tf facilitates NPs delivery across BBB [56].

## 5. Polymer Nanoparticles for Brain Cancer Therapy

Polymer NPs are solid colloidal particles that can be utilised as carriers in which the therapeutic drugs or other active components are dissolved, entrapped, encapsulated, or adsorbed on the surface of the polymer matrix [69]. The structure of the polymer NPs can range from nanospheres to nanocapsules depending on the preparation procedure. Various polymers such as chitosan, gelatin, sodium alginate, albumin and polylactides (PLA), polyglycolides (PGA), poly(lactide co-glycolides) (PLGA), polyanhydrides, polyorthoesters, polycyanoacrylates, poly(ε-caprolactone), poly(glutamic acid), poly(malic acid), poly(N-vinyl pyrrolidone), poly(methyl methacrylate), poly(vinyl alcohol), poly(acrylic acid), poly(acrylamide), poly(ethylene glycol), poly(methacrylic acid) are mostly used in nanoparticle formation for both passive and ligand- functionalized actively targeted therapy [70]. Based on the nature of drugs to be loaded and their route of administration, different synthesis methods were implemented for the production of polymer NPs that include solvent evaporation, solvent diffusion, nanoprecipitation, emulsification, reverse salting out, nano-capsules nano-precipitation, layer-by-layer (LbL) method, etc. [71,72,73]. The molecular weight, crystallinity, and stability of polymers and the drug’s physicochemical properties can be analysed to develop polymeric NPs for drug administration to the brain. Polymeric NPs have a unique ability to reach the tumour site through an active targeting route [74]. Researchers have developed docetaxel (DOC)-loaded PCL and its derivative poly (ethylene glycol)-block-poly(ε−caprolactone) methyl ether (mePEG-PCL) nanoparticles that were dispersed in a bioadhesive film and the formulation exhibited sustained release of drugs. Docetaxel-loaded nanoparticles induced more significant cytotoxicity than free docetaxel for glioma treatment [75]. The study reveals that glycopeptide-engineered poly(d,l-lactide-co-glycolide (PLGA) NPs (g7-NPs) provides in vivo evidence of endocytosis of g7-NPs and transported into the endosomes, which help to cross BBB [76]. Gaudin and co-workers have demonstrated the use of convection-enhanced delivery (CED) of NPs for improved chemotherapeutic drugs to the tumour site. They successfully administered gemicitabine, a nucleoside analogue used for the wide range of solid tumours using squalene-based NPs. The study also revealed that PEGylation of the NPs with PEG dramatically improves the distribution of squalene-gemcitabine NPs in the tumours [77].

Most of the current nanomedicines approved by the FDA for clinical use for solid tumour treatment depend on the EPR effect. The enhanced permeability and retention effect or EPR effect is a feature that allows small sized nanoparticles and other active molecules or drugs to pass due to large pore size through leaky vasculature and accumulate in the tumor location.

The brain endothelial cells and glioblastoma cells generally overexpressed a number of receptors, including the low-density lipoprotein receptor, IL-13 receptor, transferrin receptor (TfR), and nicotine acetylcholine receptor that used as drug delivery targets in the brain [78]. Numerous in vivo studies revealed that polymer NPs could circulate for a longer time and accumulate in the tumour site. It is possible to enhance the retention and accumulation of these useful NPs by decorating NPs with tumour-homing ligands such as peptide, aptamer, polysaccharides, saccharides, antibodies, flic acids, etc. [79]. Recently, pluronic micelles (PEG-PPG-PEG) have evolved as perfect candidates for brain therapy, as they can easily cross the BBB and prove their ability to inhibit drug efflux [17]. For instance, Sun et al. developed TfR-T12 peptide-modified PEG-PLA polymer nanoparticle micelles loaded with paclitaxel (PTX) for glioma therapy. They found that the polymeric micelles (TfR-T12-PMs) could be absorbed by tumour cells, cross across BBB monolayers, and inhibit the proliferation of U87MG cells in vitro. A better antiglioma effect with a prolonged median survival of nude mice-bearing glioma was also observed in comparison with unmodified PMs [80]. This suggests that TfR-T12 peptide-modified micelles can cross the BBB system and target glioma cells. In Table 3 and Table 4 are shown various synthetic and natural polymer-based NPs for GBMs therapy or diagnosis.

Recently, much research has been carried out on combined photo-based therapy along with other conventional therapy or imaging for glioblastoma treatment. For example, a novel photoacoustic and photothermal guided semiconducting polymer nanoparticles (SPNs) using poly (ethylene glycol)-block-poly (propylene glycol)-block-poly (ethylene glycol) (PEG-b-PPG-b-PEG) and SP were reported. The SPNs displayed efficient cellular internalisation for PAI and PTT toward U87 cells and accumulated in subcutaneous as well as brain tumours upon intravenous injection and induced efficient cell death upon NIR-II light irradiation [81].

The recent updates reveal that conjugated polymer nanoparticles (CPNs) are performing well as photosensitiser (PS) in photodynamic therapy (PDT). This efficiency is achieved by CPNs due to their uniform size, biocompatibility, and outstanding ROS production due to extraordinary photo-physical properties as well as fluorescence emission. It is found that porphyrin doped CPNs can eliminate GBMs through ROS-induced apoptotic damage [82].

## 6. Polymer Nanoparticles in the Diagnosis of Brain Cancer

**Figure 3:**
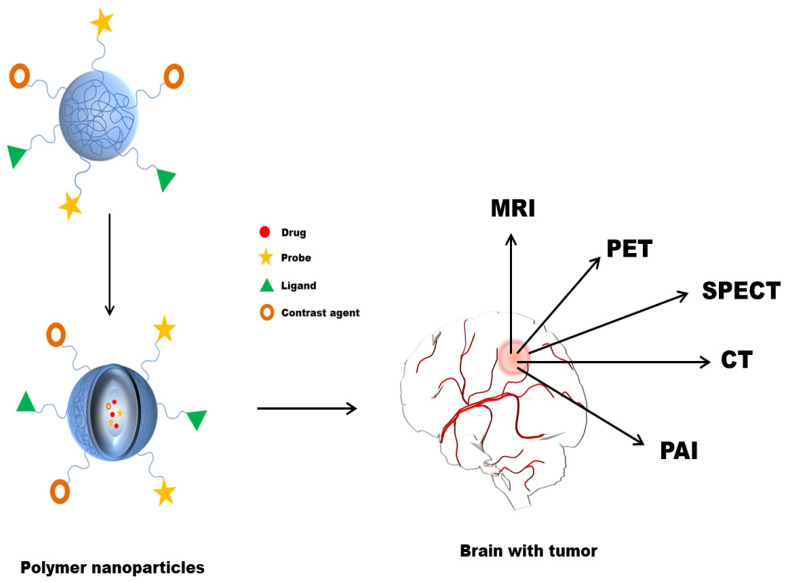
Polymer NPs in imaging for improved diagnosis of brain cancer.

Before surgery, a high-resolution image using imaging modalities is required for glioma detection. Owing to the invasiveness of glioma cells, determining the exact tumour boundary by eye is challenging. Proper imaging of a tumour is essential for assessing the extent of tumour distribution before surgery and the response to a treatment regimen after surgery [5]. Several available techniques for visualisation and diagnosis of brain cancer glioma include optical and ultrasound (US) imaging, photoacoustic (PA) imaging, computed tomography (CT), positron emission tomography (PET), single-photon emission computed tomography (SPECT) and fluorescence (FL) imaging techniques (Figure 3) [112]. Currently, magnetic resonance imaging (MRI), a non-invasive technique that can detect the size, shape, and tumour location, is initially employed diagnostic method for patients with suspected GBM [113]. MRI can determine the boundaries of the tumour tissues and/or intraoperative to elucidate tumour outline during surgical resection by applying gadolinium (Gd). Due to a shorter half-life, Gd must be administered often to maintain blood levels for efficient scanning. The use of intraoperative ultrasonography to obtain integrated brain tissue imaging is another non-optical method. However, this approach does not provide enough information for detecting smaller or superficial brain tumours. Other invasive techniques for analysing brain tumour tissues include Raman spectroscopy, optical coherence tomography, fluorescence spectroscopy, and thermal imaging [114]. Computed tomography (CT) can also be used to determine the presence of the tumour. Still, its use is relatively lesser in clinics for diagnosing GBM due to poor resolution compared to MRI [115]. Likewise, positron emission tomography (PET) imaging with 11C-methionine could be an effective diagnostic tool for GBM patients’ prognosis [116,117]. To understand cancer tumours, precise preoperative imaging and painless sensitive post-imaging techniques to provide real-time data are demanded. Current imaging modalities, however, lack accuracy, sensitivity, and specificity. Nanotechnology has sparked interest in bioimaging and biosensing in recent years.

‘Nanodiagnostics’ combined with nanotechnology could provide a drug delivery system with traditional diagnostic and imaging procedures [118,119]. Nanotechnology has made it easier to acquire data with great precision and accuracy while avoiding invasive procedures. NPs with tunable optical, magnetic, and electrical properties are able to provide diagnostic tools for detection and imaging brain cancer/tumours [120]. Biocompatible NPs owing ideal physical characteristics, such as surface chemistry, morphology, solubility, stability, etc., facilitate drug delivery and imaging as it acts as image contrast agents [121]. Polymer NPs could be a good reservoir system for drugs and a platform for additional modification for efficient tumour targeting or imaging [122]. Polymer NPs possess various advantages in drug delivery to the brain that can entrap or carry drugs that prevent them from metabolism and excretion. Moreover, NPs can easily transport drugs across the BBB without changing the barrier properties [31,123,124]. In this section, polymer NPs utilised in the diagnosis and detection of brain cancer glioma until now are primarily focused. The imaging and diagnosis techniques currently being investigated with reference to polymer NPs are listed in Table 5.

Polymer-based superparamagnetic NPs have mainly been employed as drug delivery systems and contrast agents in MRI imaging. These NPs are highly stable and biocompatible, can prolong systemic circulation time, have drug loading ability and control of drug release, and combine with their magnetic performance for MRI [125]. Ganipineni et al. synthesised paclitaxel (PTX) and superparamagnetic iron oxide (SPIO)-loaded PEGylated PLGA-based NPs (PTX/SPIONPs) and analysed for therapeutic efficacy in an orthotopic U87MG model. The cellular internalisation of these NPs was found to be concentration dependent. The MRI scanning displayed the blood–brain barrier disruption in the glioma affected location. Moreover, enhanced accumulation was also observed in ex vivo bio-distribution analysis of GBM-bearing mice with magnetic targeting [126]. Researchers have evaluated SPIO-loaded brain penetrating PLGA NPs by CED administration on rat models and visualised using positron emission tomography (PET) and MRI [127]. SPIO-loaded NPs showed excellent transverse (T2) relaxivity. After CED of NPs, the biodistribution in the brain was analysed using MRI, which revealed a period of one month longer signal attenuation of SPIO-loaded brain-penetrating PLGA NPs. The co-administration of SPIO-loaded PLGA NPs allows intraoperative monitoring of biodistribution in the brain in order to ensure the delivery to tumour location and therapeutic effect over time [127]. Researchers have developed Polysorbate 80 coated temozolomide-loaded PLGA-based superparamagnetic nanoparticles (P80- TMZ/SPIO-NPs), evaluated for anti-glioma activity and analysed as a diagnostic agent for MRI [128]. The superparamagnetic P80-TMZ/SPIO-NPs showed a significant antiproliferative effect and remarkable cellular internalisation on C6 glioma cells. Moreover, the in vitro MRI scanning revealed that P80-TMZ/SPIO-NPs could also serve as a good contrast agent [128].

## 7. Limitations and Challenges

**Figure 4:**
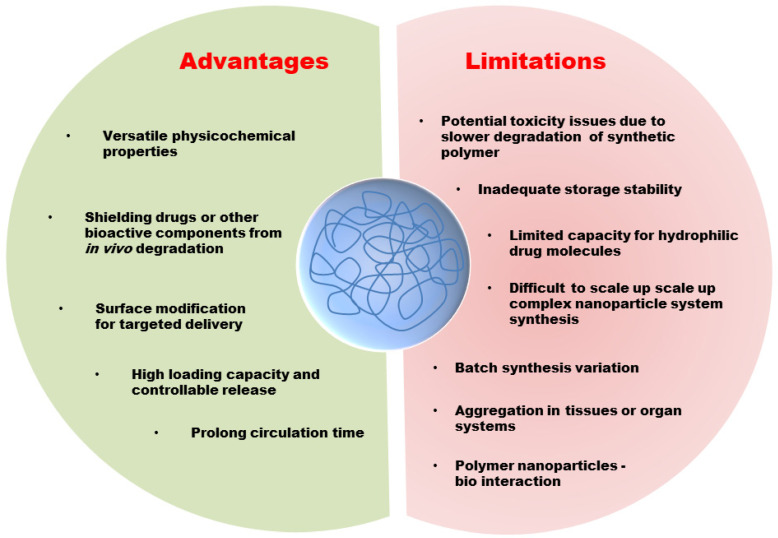
The advantages and limitations of polymer NPs in drug delivery and therapy.

From the past times, tremendous developments have been evidenced in brain cancer therapy. Yet, there have not been emerged significant changes in mortality rate and improving patients’ quality of life. Although nanoparticle-based drug delivery systems have brought a new horizon, many challenges remain and need to be solved in the future. The development of effective polymeric NPs for drug delivery and targeting is a challenging task for clinical translations. The advantage and limitations are summarised in Figure 4. The toxicity of these systems is one of the main challenges. The slow degradation rate of polymer NPs induce a longer circulation time in the body and could cause unknown complications.

Further, extensive investigations are required for optimization of the NPs. One of the major obstacles in clinical translation is the interaction of NPs and biological systems. Upon entering the complicated biological system, the designed polymeric NPs will instantly interact with neighboring biomolecules, leading to the formation of protein corona that alters their properties. This affects NPs size, stability, surface properties and determines the pharmacokinetics, biodistribution, cellular internalisation, intracellular trafficking, immune system, and toxicity [133,134,135,136]. In addition, more in vitro and in vivo studies are required to better understand the mechanisms in targeted nanoparticle-based therapy. Several essential factors related to the in vivo behaviour of NPs and their effect on other healthy brain cells are hence required to be extensively examined. Currently, there is still insufficient pre-clinical data of polymer-based NPs on brain delivery, data to correlate in vitro-in vivo observation, which makes it difficult to conclude about their therapeutic efficacy.

## 8. Future Perspective and Conclusions

Glial originated brain cancers are the most aggressive gliomas that depict a threat to humans. The conventional therapies are still inefficient to overcome due to tumor heterogeneity and, specifically, the blood–brain barrier (BBB) of malignant gliomas. The polymeric nanoparticles-based brain cancer therapy approaches are currently gaining interest due to the drug safety, controllable drug release, and efficient targeting in tumors. Most importantly, reports revealed that polymer NPs could even transport across BBB. In this review article, we summarize the newest breakthroughs in the use of polymer nanocarriers for drug delivery, therapy and diagnosis of brain cancer are explored, emphasizing how they are a critical aspect of modern anticancer drug delivery strategies. Various polymer NPs have been generated to reduce anticancer drug losses, premature degradation, enhance drug availability, and reduce drug toxicity by improving drug accumulation in specific organs and tissues. Although the potential impact of polymer NPs in cancer therapy is exceedingly promising, numerous obstacles that currently limit their widespread clinical usage must be solved. For polymer NPs to be used in clinical trials, long-term safety investigations must be conducted in various animal models to eliminate the possibility of non-endogenous components accumulating in the body causing any harm. As a result, huge costs must be provided when conducting in vivo pharmacokinetic studies to evaluate the applicability in the human body. Another factor to consider is the challenges that may arise when transitioning from laboratory to large-scale production. The scaling up of the preparatory process is a major obstacle that must be surmounted. A significant number of polymer NPs are currently in the pre-clinical stage of development, but only one system has entered a clinical study. This is primarily because several challenges impede further development, such as a lack of potency in animal models and toxicity concerns. To overcome the aforementioned concerns, researchers need to focus more on new therapeutic innovations such as revising fabrication processes to modify and improve polymeric NPs in order to accommodate the demand for various anticancer drugs for effective clinical feasibility. New therapeutic innovations also include novel therapeutic strategies for combination therapy and stimuli-activated drug delivery. For example, delivering two or more anticancer drugs simultaneously might enhance the treatment of various cancer developments by targeting different tumour related signalling pathways, resulting in a synergistic therapeutic impact. In addition, the researcher needs to improve the targeting of cancer stem cells (CSCs) for effective cancer therapeutic effect as CSCs is a critical factor for tumour recurrence. In conclusion, pre-clinical experimentation and clinical trials are mandatory for an efficient polymer nanoparticle-based anticancer therapy. Hopefully, all of these developments will lead to more patient-specific and targeted anticancer therapies.

## Figures and Tables

**Table 1:** Advantages and limitations of conventional glioblastoma therapy.

Conventional Therapy	Advantage	Limitation
Resection	Local removal of a tumour	Entire tumour cannot be removed GBM cannot be fully cured, may relapse within 2 to 3 cm of the original tumour boundary Invasive in nature
Radiotherapy	Standard treatment protocol for HGGs	Necrosis of normal brain tissue Neuronal damage Resistance to radiation of tumour cells
Chemotherapy	Standard therapy for cancer, cytotoxicity	High dose BBB Low accumulation of the drug Tumour heterogeneity Resistance to drug

**Table 2:** Summary of BBB permeability based on polymer-based nanoparticles.

Polymer Nanoparticles	Cargo	Internalisation Mechanism	Cell Line/Animal Model	Remarks	References
Trimethylated chitosan (TMC)-modified PLGA NPs	Coenzyme Q10 6-coumarin	AMT	SH-SY5Y cells, AD transgenic mouse brains	Increase uptake of PLGA nanoparticles, neuroprotective effects of Q10 observed in TMC-PLGA NPs than PLGA-NP.	[57]
Angiopep-2 modified PLGA NPs	Doxorubicin (DOX), Epidermal growth factor receptor (EGFR) siRNA	RMT	U87MG cells, brain orthotopic U87MG glioma xenograft model	Improved DOX and siRNA cellular uptake, NPs able to cross BBB.	[58]
Lactoferrin, folic acid modified PLGA NPs	Etoposide	RMT	HBMEC/HA monolayer, U87MG cells	PLGA NPs cross BBB and enhanced 2-fold uptake with Lf-and FA.	[59]
RVG29 modified PLGA NPs	Docetaxel	RMT	C6 cells, bEnd3 monolayer BBB model	Better BBB penetration in vitro.	[60]
OX26 Mab modified PLGA NPs	Temozolomide (TMZ)	RMT	U215 and U87, in vitro HBLECs monolayer model	Improved TMZ internalisation in glioblastoma cells.	[61]
T7- modified, magnetic PLGA nanoparticulate system (MNP/T7- PLGA NPs)	paclitaxel (PTX) and curcumin (CUR)	RMT	U87 cells and mouse brain endothelial cell line bEnd.3., mice bearing orthotopic glioma (U87-Luc)	>10-fold increase in cellular uptake studies and a >5-fold enhancement in brain delivery compared to the non-functionalized NPs.	[62]
Angiopep conjugated PEG-PCL nanoparticles (ANG-PEG-NP)	paclitaxel (PTX)	RMT (LRP-mediated transcytosis)	U87 MG, Male BALB/c nude mice and ICR mice	The penetration, distribution, and accumulation into 3D glioma spheroid and in vivo glioma region of ANG-PEG-NP was higher than that of plain PEG-PCL nanoparticles (PEG-NP).	[63]
dCatAlb encrusted DOX-loaded PLGA nanoparticle	Doxorubicin (DOX)	AMT	monolayer bEnd.3 cells	Enhanced BBB permeation	[64]
cRGD/PEG-SS-PCL micelles	Doxorubicin (DOX)	RMT	U87MG glioma xenografts	Efficient accumulation	[65]
DOX-loaded cRGD-SS-NGs	Doxorubicin (DOX)	RMT	U87-MG cells, U87-MG glioblastoma xenograft in nude mice	Facilitated cellular uptake and intracellular DOX release	[66]
T7–PEG–PLGA micelles	Carmustine (BCNU)	RMT	U87-MG cells, BALB/c nude mice	Accumulation in tumour more efficiently than unconjugated one	[67]
PLGA based SSTR2 pep-DIM-NPs	3,3′-diindolylmethane	RMT	C6 glioma cells, rat Glioma model	Accumulation of the NPs into rat brain tumour sites by crossing the BBB	[68]
L-carnitine modified PLGA nanoparticles (LC-PLGA NPs)	Taxol and paclitaxel (PTX)	CMT	hCMEC/D3, T98G cells	Efficient accumulation	[46]

Abbreviation: Adsorption-mediated transcytosis (AMT), carrier-mediated transport (CMT), and receptor-mediated transcytosis (RMT).

**Table 3:** Synthetic polymer-based nanoparticles for brain cancer glioma therapy.

Polymers	Method of Preparation	Therapeutic Drug/Other	Targeting Receptor/Molecule	Diagnostic Component	Cell Line/Animal Model	Remark	References
Synthetic protein nanoparticle (SPNP)	Electrohydrodynamic (EHD) jetting	siRNA	STAT3i	Alexa Fluor 647- labeled albumin	GL26 syngeneic mouse glioma model	Five-fold increase in iRGD loaded SPNP in glioma cell observed in comparison to NPs without iRGD. A total of 87.5% of mice developed anti-GBM immunological memory.	[83]
Porphyrin doped conjugated polymer nanoparticles (CPNs)	Controlled nanoaggregation	-	m-RNA	DCF-DA	U-87 MG, T98G and MO59K	NPs enhance the efficacy of PDT to eliminate tumor via ROS generation. MO59K and U-87 MG cells are died with CPN having IC50 values 8 mg/L and 9 mg/L, however, T98G cells are found resistant to CNP-PDT.	[84]
PLGA	Single-emulsion, solvent evaporation technique	Paclitaxel	-	-	U87MG with rats and pigs’ model	Enhanced in vivo efficacy	[85]
PBAEs	Step-wise synthesis	DNA		Cy3 dye	BTICs from patient	More than 60% transfection efficacy is observed.	[86]
cRGD-conjugated PGNRs	Ligand exchange method	-	α_v_ β_v-_ integrin	-	U87MG	cRGD-PGNRs is proved having excellent tumor targeting ability, no cytotoxicity, and sufficient cellular uptake.	[87]
Aptamer/gold nanorod conjugate	Step-wise synthesis	Sgc8 aptamer	Cell protein	Fluorescein	Rat or mouse model	A total of 99.09% binding affinity due to the aptamers. Complete destruction of GMB on exposure to LAER is observed.	[88]
Poly(N-isopropylacrylamide)-based nanogels and magnetic NPs composite	Co-polymerisation and co-evaporation	Ferrrofluid	-	Sodium fluorescein	Rat model	The drug dose delivered to tumor site is directly proportional to the duration of the “on” pulse.	[89]
PEG−PBAE/ePBAE nanoparticles (NPs)	Step wise synthesis, Michael addition	Plasmid DNA, pHSV-tk, ganciclovir	-	Hoechst 33342 dye	GBM1A and BTIC375 cells/Mice model	PEG−PBAE/ ePBAE NP shows 54 and 82% transfection efficacies in GBM1A and BTIC375 cells while it is 37 and 66% for optimised PBAE NPs without PEG. Death of cancer cell with enhancement of mice life time was observed.	[90]
TEB	Co-precipitation	-	Transferrin (TfR), lactoferrin (LfR) and lipoprotein (LRP)	-	bEnd.3/Mouse model	Ligand-coated TEB nanoparticles are transported across BBB with high efficacy.	[91]
PEG-PLA	Emulsion/ solvent evaporation technique		Neuropilin (NRP), tLyp-1 peptide		Human umbilical vein endothelial cells and Rat C6 glioma cells	tLyp-1 peptide functionalised NPs show better performance in paclitaxel glioma therapy. Observed inhibition of avascular C6 glioma spheroids. Interestingly tLyp-1-NP-PTX formulations shows higher antiproliferation ability with IC50 0.087 mg/mL in comparison to NP-PTX and Taxol.	[92]
Transferrin modified PEG-PLA	Double emulsion and solvent evaporation method.	Resveratrol (RSV)	-	-	C6 and U87 glioma cells	RSV-conjugates decreased brain tumor volume and accumulated well in comparison to free RSV.	[93]
Polysorbate-coated NPs	Surfactant mediated ultrasonication	Doxorubicin (DOX)	-	Evans Blue solution	Glioblastoma 101/8-bearing rats	Enhanced permeability and retention effect	[94]
PCL	Solvent evaporation technique	Irinotecan hydrochloride trihydrate (IRH)	-	-	HGG cells	IRH-loaded PCL NPs has excellent anti-brain tumor activity. PCL shows better drug encapsulation than PLGA.	[95]
cRGD-directed AuNR/PEG–PCL hybrid NPs	Nanoprecipitation	Doxorubicin (DOX)		Cy7	Human U87MG glioma	Controlled release of doxorubicin into human glioblastoma using mice model is achieved that leads to inhibition of 100 % tumour growth.	[96]
PCL-Diol-b-PU/gold nanofiber composite		Temozolomide (TMZ)			U-87 MG human glioblastoma cells	Slower release of TMZ showing its high potential as implantable device for drug release. Enhanced activity against the U-87 cell.	[97]
PEG-PCL NPs conjugated with ALMWP	Emulsion/solvent evaporation method	Paclitaxel (PTX), Taxol	-	coumarin-6	C6 cells	Animals treated for C6 gliomas with ALMWP-NP-PTX survive longer than those treated with Taxol-NP-PTX.	[98]

Abbreviation: PLG: poly(lactide-coglycolide), DCF-DA: 2’,7’-dichlorofluorescin-diacetate PBAEs: poly (β-amino ester) s, cRGD: cyclic RGD peptides, PGNRs: PEGylated gold nanorods, PEG: polyethylene glycol, PSMA: prostate-specific membrane antigen, NR: nanorods, PCL-diol: poly (ε-caprolactone diol), PU: polyurethane, ALMWP: activatable low molecular weight protamine.

**Table 4:** Natural polymer-based nanoparticles for brain cancer glioma therapy.

Natural Polymer-Based Nanoparticles	Method of Preparation	Therapeutic Drug/Other	Targeting Receptor/Molecule	Diagnostic Component	Cell Line/Animal Model	Remark	References
Den-angio nanoprobe	Step-wise synthesis	-	LRP receptor-mediated endocytosis		U87MG	Den-Angio shows localisation in the brain tumours and makes image-guided tumour resection possible.	[99]
CDP-NP	Single-step synthesis at room temperature, self-assembly method	-	Proteins	e-GFP, luciferin	BV2, N9 microglia (MG) cells and GL261 glioma cells/mice model	CDP-NPs were efficiently taken up by BV2 and N9 microglia (MG) cells compared to GL261 glioma cells.	[100]
Silver NPs impregnated alginate–chitosan-blended nanocarrier	Polyelectrolyte complex formation reaction		DNA	Acridine Orange/Ethidium Bromide dual stain	U87MG	Extensive DNA damage was observed on cell cycle analysis.	[101]
Hyaluronan (HA)-grafted lipid-based NPs (LNPs)	Amine coupling strategy	rRNA interference (RNAi), doxorubicin and BCNU	CD44 receptor	DAPI (blue)	T98G, U87MG, and U251	Prolonged survival of treated mice in the orthotopic model was observed.	[102]
Cardamom extract-loaded gelatine NPs (CE-loaded GNPs)	Two-step de-solvation method	Cardamom extract	-	-	U87MG	Extract to polymer ratio as 1:20 was found to be the best with entrapment. efficiency close to 70%	[103]
NK@AIEdots (natural-killer-cell-mimic nanorobots with aggregation-induced emission)	Step-wise synthesis, assembly process	-	-	-	U-87 MG, bEnd.3	The tumour growth was also successfully inhibited by NK@AIEdots on exposure to NIR light.	[104]
Heparin-based polymer)–SWL–(cRGD) NPs (S = serine, W = tryptophan, L = leucine)	Coupling reaction		α_v_ β_v_ and EphA2 in glioma	f Oregon-green488	U87 and U251	NPs easily pass-through BBB to the tumour site. In addition, inhibition of glioma cell proliferation is noticed.	[105]
poly-L-arginine-chitosan-triphosphate matrix (ACSD)	Green co-precipitation method	Doxorubicin, SPIONs	-	Prussian blue staining and inductively coupled plasma	Rat glioma C6 cells	ACSD NPs are proved as promising theranostic formulation MRI analysis shows uptake of NPs in C6 glioma cells. There observed 38.6% drug release in neutral pH while 58% in acidic pH. A 44-fold increase in IC_50_ value of doxorubicin was found when the drug was loaded in NPs.	[106]
Albumin nanoparticles (NPs)	Two-step synthesis, grafting	Paclitaxel (PTX)	Substance P (SP) peptide	Cou-6 dye	Glioma U87 cells	Albumin nanoparticles are found satisfactory for drug delivery vehicles for the treatment of GBM. The targeting effect of SP, and efficient cellular uptake of SP-HSA-PTX NPs into brain capillary endothelial cells (BCECs) and U87 cells is improved.	[107]
Human serum albumin (HSA) NPs	High-pressure homogeniser technique	Doxorubicin	-	LysoTracker	bEnd.3 cells as well as U87MG	Anti-glioma efficacy is improved due to the dual-enhanced system of dual cationic absorptive transcytosis and glucose-transport by using c- and m-HSA together.	[108]
Albumin NPs	Green synthesis	Paclitaxel and fenretinide	-	CY5 dye	Human glioma U87, U251 cells, mouse glioma C6, GL261 cells,	The albumin-binding proteins are found to be overexpressed in the tumour/glioma cells, where epithelium cells are responsible for delivering NPs to brain tumours.	[109]
Menthol-modified casein NPs(M-CA-NP)	Self-assembled micelle formation	10-Hydroxycamptothecin, methanol	-	Cou-6	C6 cells	Resulted in enhanced drug accumulation in the tumour site.	[110]
Transferrin-functionalised NPs (Tf-NP)	Functionalisation	Temozolomide and the bromodomain		Cy5.5	U87MG and GL261 cells	Therapy showed 1.5- to 2-fold decrease in tumor burden and corresponding increase in survival in tumor bearing mice	[111]

Abbreviation: Den: dendrimer, Angio: angiopep-2, PDT: photodynamic therapy, CDP-NP: cyclodextrin-based nanoparticle, TEB: triphenylamine-4-vinyl- (P-methoxy-benzene), DAPI: 4′,6-diamidino-2-phenylindole, SPIONs: superparamagnetic iron oxide nanoparticles.

**Table 5:** Polymer nanoparticles in imaging and diagnosis of brain cancer therapy.

Nanoparticles	Detection Method	Cell Line	Animal Model	Therapy/Drug	References
SPIONs and DOX loaded poly-l-arginine-chitosan-triphosphate matrix (ACSD) NPs	MRI	C6 glioma cells	-	DOX	[106]
P80- TMZ/SPIO-NPs (PLGA coating)	MRI	C6 glioma cells	-	TMZ	[128]
Micelles SPION and Au NPs (PEG-PCL coating)	MRI, CT	-	U251 xenograft and orthotopic brain tumour models.	Radiotherapy	[129]
Chitosan-dextran superparamagnetic NPs (CS-DX-SPIONs)	MRI	C6 glioma, U87	orthotopic C6 gliomas in rats	-	[130]
DOX-Ps@80-SPIONs	MRI	glioblastoma C6 cells	Glioma-bearing rats	DOX	[131]
Paclitaxel (PTX) and superparamagnetic iron oxide (SPIO)-loaded PEGylated poly (lactic-*co*-glycolic acid) (PLGA)-based NPs(PTX/SPIONPs)	MRI	-	orthotopic U87MG model	PTX	[126]
SPIO-loaded brain penetrating PLGA NPs	PET, MRI	-	rat model	-	[127]
[^18^F] NPB4-labeled and C6-loaded PLGA NPs	PET	-	rats bearing BCSC-derived xenografts	-	[85]
TMZ and iron oxide-containing polymer NPs(PMNPs)	MRI	U87 glioma cells	rodent model	TMZ	[132]

Abbreviation: *N*-(4-[^18^F] fluorobenzyl) propanamido-PEG_4_-Biotin, brain cancer stem cells (BCSCs).

## Data Availability

The study did not report any data.
